# Efficient and Continuous Carrier-Envelope Phase Control for Terahertz Lightwave-Driven Scanning Probe Microscopy

**DOI:** 10.1021/acsphotonics.3c00555

**Published:** 2023-10-11

**Authors:** Jonas Allerbeck, Joel Kuttruff, Laric Bobzien, Lysander Huberich, Maxim Tsarev, Bruno Schuler

**Affiliations:** † nanotech@surfaces Laboratory, Empa, Swiss Federal Laboratories for Materials Science and Technology, Überlandstrasse 129, 8600 Dübendorf, Switzerland; ‡ Department of Physics, University of Konstanz, Universitätsstrasse 10, 78464 Konstanz, Germany

**Keywords:** ultrafast scanning tunneling microscopy, THz-STM, ultrafast photocurrents, frustrated total internal reflection, field-driven tunneling, photoemission sampling

## Abstract

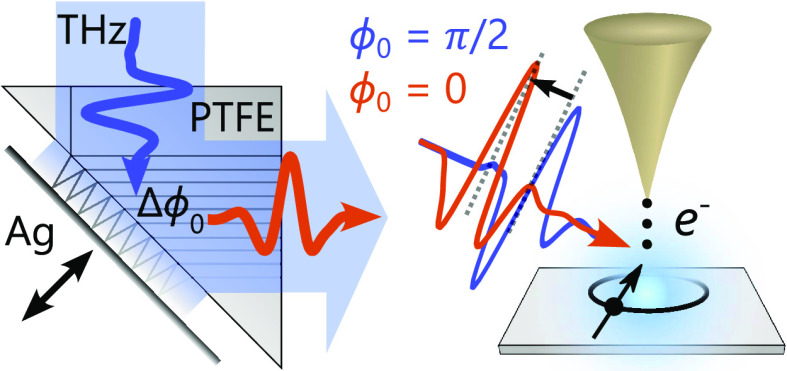

The fundamental understanding of quantum dynamics in advanced materials requires precise characterization at the limit of spatiotemporal resolution. Ultrafast scanning tunneling microscopy is a powerful tool combining the benefits of picosecond time resolution provided by single-cycle terahertz (THz) pulses and atomic spatial resolution of a scanning tunneling microscope (STM). For the selective excitation of localized electronic states, the transient field profile must be tailored to the energetic structure of the system. Here, we present an advanced THz-STM setup combining multi-MHz repetition rates, strong THz near fields, and continuous carrier-envelope phase (CEP) control of the transient waveform. In particular, we employ frustrated total internal reflection as an efficient and cost-effective method for precise CEP control of single-cycle THz pulses with >60% field transmissivity, high pointing stability, and continuous phase shifting of up to 0.75 π in the far and near field. Efficient THz generation and dispersion management enable peak THz voltages at the tip–sample junction exceeding 20 V at 1 MHz and 1 V at 41 MHz. The system comprises two distinct THz generation arms, which facilitate individual pulse shaping and amplitude modulation. This unique feature enables the flexible implementation of various THz pump–probe schemes, thereby facilitating the study of electronic and excitonic excited-state propagation in nanostructures and low-dimensional materials systems. Scalability of the repetition rate up to 41 MHz, combined with a state-of-the-art low-temperature STM, paves the way toward the investigation of dynamical processes in atomic quantum systems at their native length and time scales.

## Introduction

Coherent control of ultrafast electromagnetic waveforms is crucial to understanding and manipulating fundamental carrier dynamics. Single-cycle laser pulses have become an established tool to control transport and tunneling processes with extreme precision and high time resolution.^1−4^ At the same time, nanoscale spectroscopic probes have united subpicosecond time and atomic spatial resolution, enabling the investigation of carrier dynamics in atomic and molecular systems.^1,5^ On the forefront of these technological advancements, the terahertz (THz) lightwave-driven scanning tunneling microscope (THz-STM) opened up new horizons to study electronic,^6−8^ vibronic,^1,9^ and excitonic^10,11^ dynamics at unprecedented resolution. The low frequency and off-resonant character of THz pulses can easily reach the regime of field-driven tunneling, where the electric field acts as a transient voltage pulse across a conductive nanojunction with negligible thermal impact.^12^ The advancement of commercial laser technology at multi-MHz repetition rates,^7,13^ optical systems for generating carrier-envelope phase (CEP)-locked THz waveforms,^14−20^ and commercially available low-temperature STMs with optical access^21^ have helped to expand this new field.

Precise control of the THz waveform profile can be achieved on the basis of THz waveguides,^22,23^ total internal reflection,^24,25^ the Gouy phase,^7,26−29^ or metamaterials.^30^ However, most of these implementations require active mechanical components in the THz path or are incompatible with collinear visible alignment beams. Instead, our approach relies on frustrated total internal reflection in a polymer prism using a metallic mirror to change the evanescent coupling.^31^ This concept proves as an efficient and cost-effective method for CEP control of THz pulses, enabling continuous modulation between unipolar and bipolar waveforms. It is an enabler for ultrafast scanning tunneling spectroscopy^32^ and for perspective experiments where bipolar field transients drive luminescent excitations^33^ by generating local excitons. We demonstrate how our implementation transfers to the optical near field^25,34^ by characterizing the waveform at the STM tip using photoemission sampling (PES).^35^

## THz Generation and Waveform Sampling

**Figure 1:**
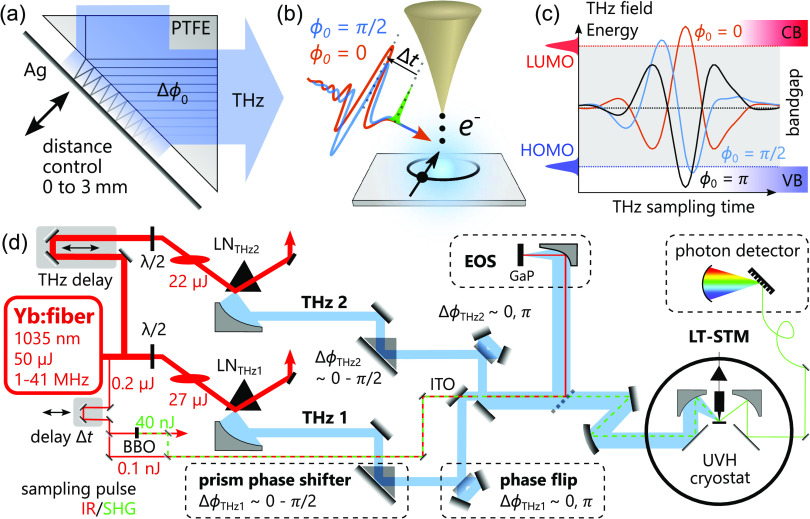
Concept and setup. (a) Metallic mirror (Ag) near the diagonal surface of a right-angle polymer prism changes the phase of a THz pulse undergoing total internal reflection. (b) Concept of field-driven tunneling and photoemission sampling of transient THz fields at the tip–sample junction. (c) THz pulses with different CEP enable state-selective tunneling in electronic states of molecules (HOMO and LUMO orbitals) or semiconductors with a conduction band (CB) and valence band (VB). (d) Optical setup as discussed in the main text. The infrared and THz beam paths are sketched in red and blue, respectively. LN: lithium niobate crystal, SH: second harmonic, EOS: electro-optic sampling, LT-STM: low-temperature scanning tunneling microscope, and UHV: ultrahigh vacuum.

We use a commercial ytterbium fiber laser (Amplitude Satsuma HP3) at 1035 nm wavelength with a variable repetition rate between 1 and 41 MHz, providing 300 fs-short near-infrared pulses with pulse energy up to 50 μJ and 50 W average power. Multi-MHz repetition rates are critical to improve the counting statistics in THz-STM experiments,^7^ while the pulse energy defines which electronic transitions can be accessed. Figure 1d schematically illustrates the optical path for infrared and THz pulses from the laser to the STM. We split the beam into a pump, probe, and sampling arm in the infrared with individual mechanical delay stages allowing up to 2 ns relative time delay. Single-cycle THz waveforms are generated via tilted-pulse-front optical rectification in two lithium niobate (LN) crystals on the basis of the geometry implemented by Wulf et al.^17,36^ using a dielectric transmission grating and two imaging lenses.

**Figure 2:**
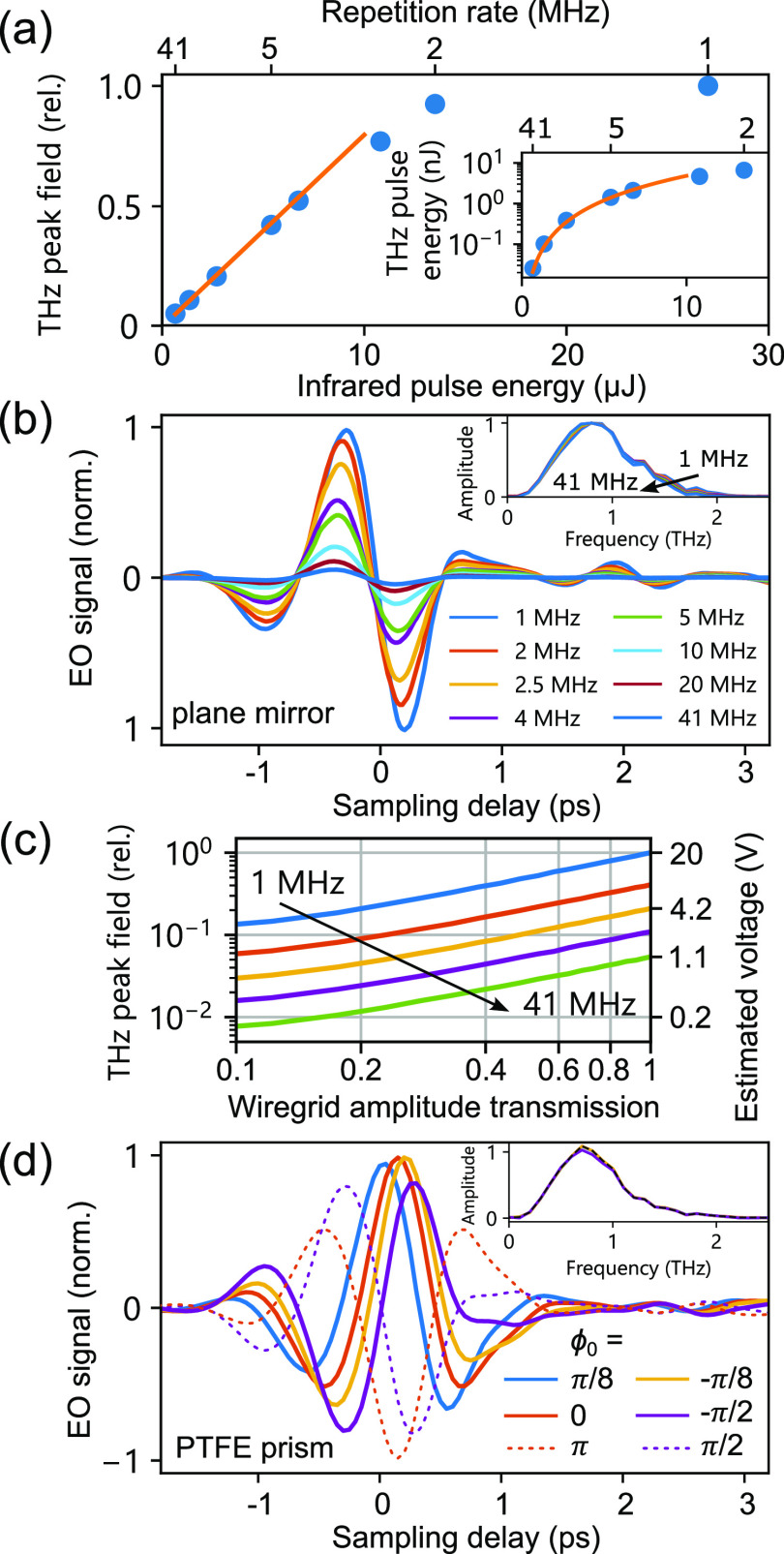
Amplitude and phase control of the THz waveform measured in the far field. (a) Infrared pump to THz conversion efficiency. The THz peak field scales linearly with pump energies up to 10  μJ. The inset shows the corresponding THz pulse energies measured directly after THz generation and collimation. (b) Electro-optic sampling (EOS) of single-cycle THz pulses generated by optical rectification in lithium niobate (LN) at multi-MHz repetition rates. A slight red shift of the THz spectrum at high repetition rates relates to an increase of the fundamental laser pulse duration. (c) THz field amplitude control enabled by a pair of wiregrid polarizers and different repetition rates: 1, 5, 10, 20, and 41 MHz. A second axis shows an estimate for the peak far-field amplitude at the STM tip calibrated at 1 MHz. (d) Continuous CEP control between 0 and π/2 enabled by frustrated total internal reflection off the polymer prism. An additional π phase flip can be achieved by adding one additional mirror to the beam path.

We reliably achieve a conversion efficiency of 6 × 10^–4^ as measured with a THz bolometer behind the LN crystal without active cooling of the crystal. Our THz pulses have a center frequency of 0.8 THz spanning a bandwidth from 200 GHz to 1.7 THz. The THz peak field scales linearly with infrared pulse energy up to approximately 10 μJ (Figure 2a), corresponding to a second-order nonlinear generation process. Figure 2b shows field transients at various repetition rates as measured by electro-optic sampling (EOS) in a GaP crystal using 100 μW of the fundamental infrared beam and a balanced photodetector. At higher infrared pulse energies, the THz pulse energy saturates due to THz reabsorption in the LN crystal by phonons or hot carriers. At 27 μJ infrared pulse energy (1 MHz), we measure a far-field THz amplitude of 3.3 kV/cm along a similar optical path accounting for the transmission losses into the ultrahigh vacuum (UHV) chamber outside the STM (see the Methods Section). A slight red shift of the THz amplitude spectrum at high repetition rates relates to an increased fundamental laser pulse duration, reducing the THz generation efficiency and damping the electro-optic signal at frequencies >0.8 THz.^37^

THz pump and probe pulses follow parallel paths, enabling independent pulse shaping, modulation, and amplitude control. Both arms are recombined in a noncollinear geometry to avoid reflections by THz beam splitters. The intrinsic THz divergence along the 2.8 m paths required for flexible pulse shaping and guidance toward the STM is compensated by considerate THz beam management with several refocusing points, as detailed in the Supporting Information. Nevertheless, divergence and absorption losses for THz pulses throughout the setup remain unavoidable. With all optical components, the power transmission from THz generation to the STM tip is approximately 2 × 10^–2^. We characterize the THz waveform in the far field outside the STM with EOS using a 1 mm-thick GaP crystal, and in the near field by photoemission sampling (PES) at the tip–sample junction using 2.4 eV (518 nm) sampling pulses obtained by second-harmonic generation of the fundamental laser. Further details can be found in the Methods Section and the Supporting Information.

Our low-temperature STM is a commercial design by Createc GmbH with optical access from two sides using a high numerical aperture (NA = 0.35) 60° off-axis parabolic mirror for precise coupling of optical pulses to the tip–sample junction.^21^ In addition to incident laser pulses, our system allows to collect optical luminescence^38^ with a sensitive spectrometer or single-photon counter using a second parabolic mirror on the opposite side. All incoming pulses are polarized along the tip axis to maximize their coupling efficiency^35^ and the near-field sampling experiments are performed in UHV conditions at 10^–10^ mbar. A low-noise current preamplifier with 10^10^ A/V mounted to the helium bath cryostat enables tunneling current detection at as low as 100 fA.

## THz Amplitude and Phase Control

Figure 2c,d shows the amplitude and phase modulation of THz pulses, enabling a complete control of the THz waveform. Precise amplitude control of THz pulses is achieved by a pair of wiregrid polarizers or by rotating the polarization of infrared pump pulses against the optical nonlinearity of the LN crystal. Alternatively, the laser repetition rate can also be increased, thereby downscaling the available THz amplitude by more than 2 orders of magnitude (Figure 2c) while simultaneously increasing counting statistics of the experiment. We note that the peak voltage estimate for high repetition rates in Figure 2c relies on amplitude scaling measured in the far field; however, we confirm similar values by independent THz amplitude calibration in the tunneling regime (see Figure S6).

The key element enabling continuous phase tuning of the THz transient is a right-angle polymer prism where the THz pulse undergoes total internal reflection (Figure 1a). By placing a metallic mirror behind the total reflection plane of the prism, we can change the dielectric environment of the evanescent THz wave, leading to a CEP shift of the THz pulse. With this simple design involving a single mechanical axis, we achieve robust and continuous THz CEP phase control exceeding π/2 along with strong pointing stability^31^ (Figure 1a). We used 25 mm right-angle prisms made of poly(tetrafluoroethylene) (PTFE) and a transparent polymer (Zeonex). An Ag mirror approaches the diagonal prism surface with precise mechanical control of a closed-loop piezo stick–slip axis. Kinematic mounts for the prism and mirror allow precise alignment and pointing control. The THz refractive index of the prisms is *n* = 1.43 for PTFE^39^ and ranges from 1.46 to 1.52 for Zeonex, as specified by the manufacturer and reference.^40^ Both prisms have approximate power transmissivity of 50% in the THz range, corresponding to 70% transmission of the peak field. In contrast to PTFE, Zeonex has the advantage that it is transparent in the visible (*n*_vis_ = 1.53^41^) enabling the use of visible alignment lasers. Figure 2d shows selected THz waveforms and their absolute CEP ϕ_o_. The relative CEP shift Δϕ is −π for a plain metallic mirror and closest to zero for the plain prism (mirror distance >2 mm). The maximum CEP shift accessible with both prisms is Δϕ_max,PTFE_ ≈ 0.75 π and Δϕ_max,Zeonex_ ≈ 0.56 π, allowing us to continuously shift the THz waveform from unipolar (ϕ_0_ = 0,π) to bipolar (ϕ_0_ = π/2) and beyond. In our measurement, we determine the absolute CEP ϕ_0_ of the THz waveform by fitting a single-cycle waveform to the data. By adding an additional metallic mirror to the THz path, we can flip the CEP in discrete steps of −π (dotted field profiles). The THz amplitude spectrum changes by less than 3% when the mirror moves 0.5 mm from the prism, which is sufficient for Δϕ > π/2. At distances greater than 1 mm, we observe a decay in the evanescent field that causes a field amplitude drop of 5–8%.

## Analytical Calculation and Numeric Simulation of the CEP Shift

**Figure 3:**
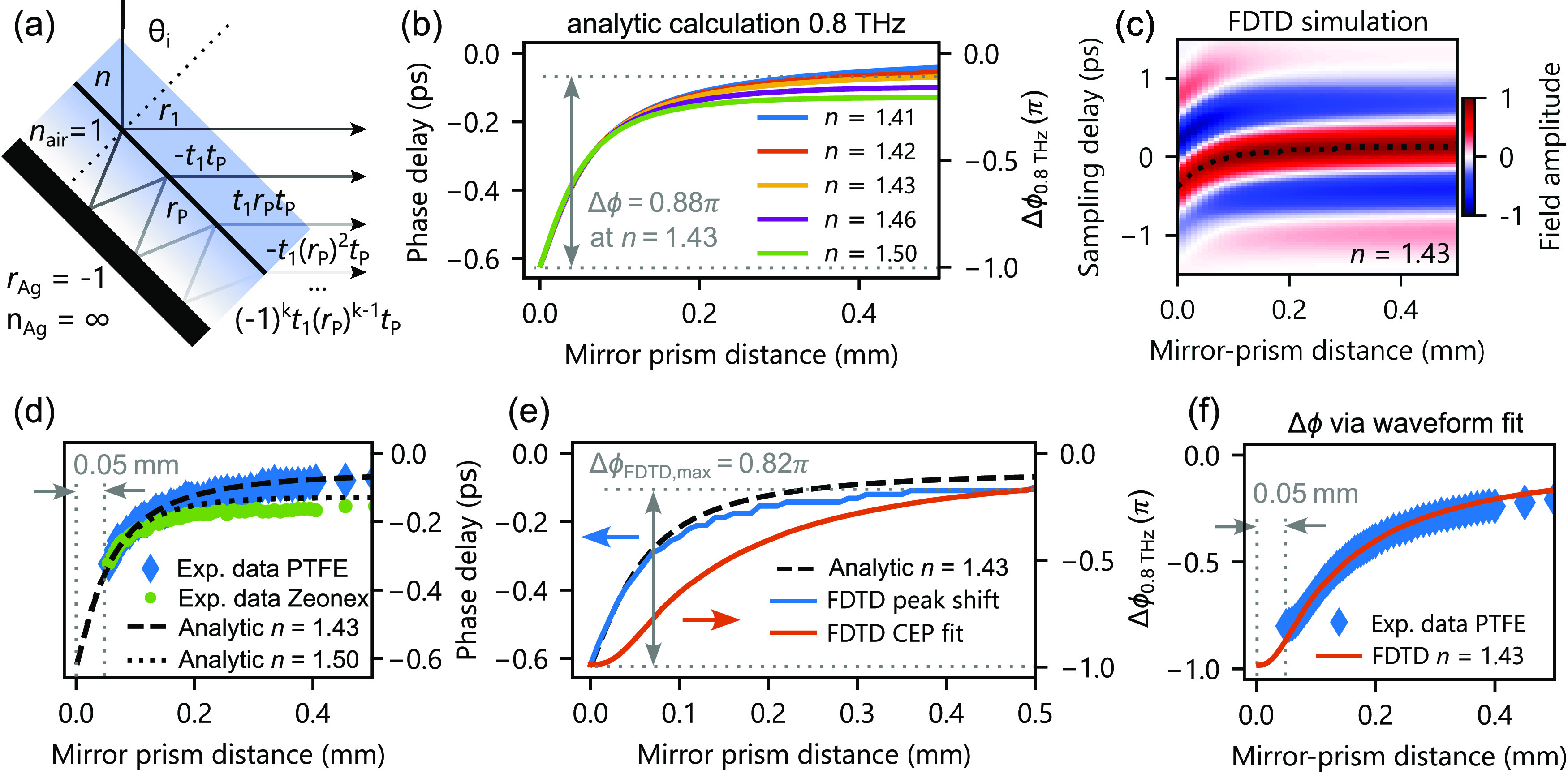
Modeling of the CEP shift. (a) Schematic of the multiple reflection model for the analytical calculation of the CEP phase shift. *r* and *t* denote Fresnel amplitude coefficients for reflection and transmission, respectively. *k* is the number of reflections at the mirror. (b) Analytical calculation of the phase delay as a function of mirror–prism distance for different refractive index materials assuming a monochromatic wave at 0.8 THz. (c) FDTD numerical simulation of the reflected THz pulse with a prism of refractive index of *n* = 1.43. The dotted line indicates the phase delay of the positive field cycle. (d) THz phase delay measured with PTFE and Zeonex prisms in the experiment and matching analytical calculations. (e) Relative phase change extracted from panel (c) using two methods: (i) tracking the peak field shift and (ii) fitting a single-cycle waveform to the data. (f) Experimental data and FDTD simulation using the CEP fit method. A 50 μm shift of the experimental data in panels (d) and (f) accounts for geometric roughness of the prism.

We use two methods to model the observed CEP shift by frustrated total internal reflection: (i) analytical Fresnel equations expressing the multiple reflections of a plane wave between the prism and metallic mirror for qualitative understanding and (ii) finite difference time domain (FDTD) simulations of a single-cycle pulse, which quantitatively reproduces the experimental CEP shift. In the analytical model, we consider an infinite sum of multiple reflections between the prism surface and the metallic mirror (Figure 3a) and estimate the absolute phase delay of a plane wave at 0.8 THz as a function of mirror–prism distance (Figure 3b). This phase delay manifests as the temporal shift of a field maximum, which is a minimum at infinite distance and −0.6 ps when the prism and metallic mirror touch. Due to small shifts of the pulse envelope when the mirror interacts with the evanescent wave, a plane wave cannot fully reproduce the CEP; however, the phase delay can be matched to the peak field delay induced by a CEP shift at 0.8 THz. The analytical model allows one to easily infer the attainable phase control from variation of the available parameters. In particular, a higher refractive index and hence smaller critical angle for total internal reflection reduces the total phase delay achieved with the system (Figure 3b). Hence, larger CEP shifts are attainable with a lower refractive index or smaller angle of incidence. Our right-angle prism requires a critical angle <45° for total reflection defining a lower bound of *n* = 1.42 for the prism material. PTFE has a close to ideal dielectric constant in the lower THz range, approaching the maximally attainable CEP phase shift from 0 to −π.

Finite difference time domain (FDTD) simulations reproduce the THz CEP shift with single-cycle pulses similar to our experimental configuration (Figure 3c). At a short distance (<0.5 mm), the metallic mirror interacts with the evanescent wave of the THz pulses, reducing the temporal delay of the reflected pulse envelope. For this reason, an accurate measure of the actual CEP must be obtained through waveform fitting instead of tracking only the absolute phase delay (Figure 3e). Both models reproduce a −π phase shift at zero distance, corresponding to a reflection at the metallic surface. Despite the plane wave approximation, our analytical calculation matches the phase delay extracted from the FDTD simulation (dotted line in Figure 3c) and the experiment (Figure 3d) well, with a slight overestimation at a larger distance. Figure 3f compares experimental data of the CEP shift measured with a PTFE prism and the FDTD simulation extracted by fitting the waveform at every distance step (Supporting Information). The kink of the CEP shift at <50 μm is due to a dominant interaction of the metallic mirror with the evanescent wave at mirror–prism distances <200 μm.

Due to fabrication tolerances (flatness) of the prism, the experiment cannot achieve a zero mirror–prism distance; hence, the −π phase shift remains practically unattainable. To match the experimental values to our theoretical model, we assume a 50 μm offset that accounts for the imperfect contact of the polymer and metal surface. This correction may also include deviations due to the prism geometry, THz divergence, large bandwidth, or polarization effects, all of which would slightly reduce the performance.

## Near-Field THz Waveform

**Figure 4:**
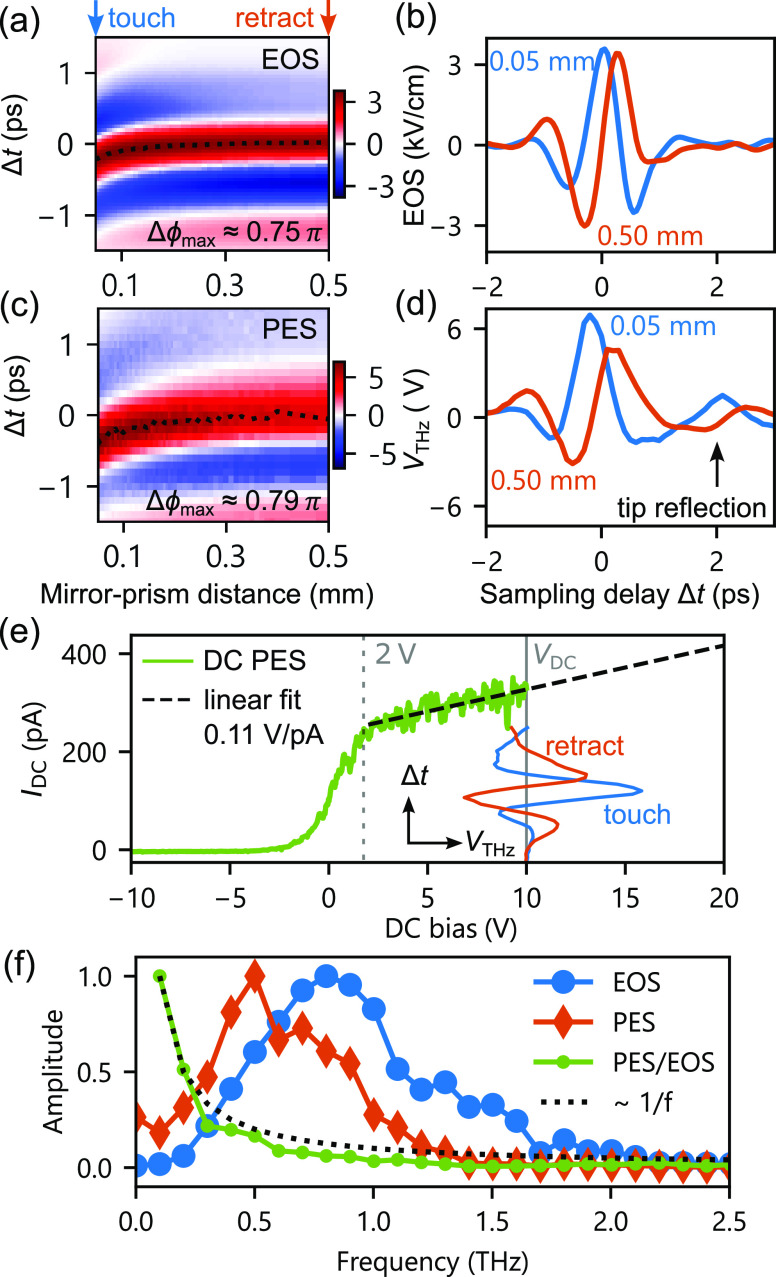
THz CEP control at the STM tip. (a–d) THz waveform measured as a function of mirror-prism distance in the far field (EOS) and in the optical near field at the STM tip measured by photoemission sampling (PES). The dotted line in panels (a) and (c) indicates the phase shift of the electric field as a guide to the eye. (e) Current–voltage characteristic of photoemission sampling, providing a linear calibration estimate for the THz field indicated by the dashed line. The total THz field was attenuated to 30% so that peak voltages remain in this linear regime. For all PES measurements, the 517 nm gate pulse intensity at the tip was approximately 4.5 nJ (1 mJ/cm^2^ at a 25 μm spot size). (f) Amplitude spectra of the THz waveform in the far and near field and the amplitude transfer function (PES/EOS amplitude ratio) showing 1/*f* scaling (dotted line) shown as a guide to the eye. All curves in panel (f) are normalized to unity.

Importantly, for the application in THz-STM, the phase control is preserved in the near field at the STM tip apex, as demonstrated by photoemission sampling (PES)^6,35^ at a few-100 nm tip–sample distance. Figure 4a–d shows our CEP control of the THz waveform in the far and near field, demonstrating almost identical CEP cycling performance as a function of mirror–prism distance. To characterize the THz waveform at the tip–sample junction, we employ PES using 2.4 eV pulses generated as the second harmonic of a fraction of the fundamental laser pulses in a 1 mm-thick beta-barium borate (ß-BBO) crystal. Our sampling pulses with few-nJ pulse energy and a temporal duration of <300 fs are overlapped with the THz pulse using the same indium tin oxide (ITO) mirror used for EOS and focused to the STM tip, collinear with the THz beam. We use an electrochemically etched tungsten tip with a gold-coated apex hovering approximately 1 μm above the Au(111) sample during PES measurements. Due to tight focusing of the parabolic mirror, few-nJ pulse energies at the STM tip lead to photoemission currents of few-100 pA at 10 V tip–sample bias.

Figure 4e shows the dc current–voltage (*I*–*V*) characteristics with linear progression above +2 V. In the linear regime around *V*_dc_ = 10 V, there is a one-to-one correspondence between the applied voltage and measured current. In the PES measurement, we sample the tunneling current only in a very short time window where the 517 nm gate pulse is impinging on the tip. In this way, the THz voltage transient can be considered quasistatic such that the dc bias and THz voltage at a specific time delay add. Thus, the induced THz voltage directly translates to a current modulation that maps the THz waveform when scanning the delay of the sampling pulse. The dc PES signal also provides an estimate for the absolute THz voltage given by the linear scaling factor.^35^ Due to dependences on sampling beam alignment, tip and sample material, tip–sample distance, and thermal drift, this method serves only as an order of magnitude estimate.

Figure 4f shows the normalized amplitude spectra of EOS and PES THz transients and the corresponding transfer function (ratio of PES and EOS amplitude spectra) with approximate 1/*f* scaling related to the antenna geometry that strongly enhances lower frequencies.^35^ As a result, the center frequency of the THz waveform decreases from 0.8 THz in the far field to 0.5 THz in the optical near field at the tip. For a metallic tip and sample, the THz near-field enhancement can be on the order of 10^3^ to 10^6^ according to previous estimates.^5,6,25,29,34,42,43^ THz pulse reflections from edges along the tip shaft are responsible for tailing field cycles of the near-field waveform (Figure 4d), as also seen by the spectral dip of the PES near-field spectrum at 0.6 THz in Figure 4f. This remains an extrinsic feature of the specific mesoscopic tip shape, which can be optimized by chemical etching protocols.^6^ By coincidence, the absolute phase of far- and near-field waveforms in our experiment (Figure 4b,d) is similar, indicating that the Gouy phase^28^ and near-field enhancement,^34^ i.e., the tip geometry,^25^ compensate each other. We note that at high PES gate pulse powers and large THz-modulated currents, waveform distortions and amplitude variations due to space charging must be considered.^35^ Future THz-STM measurements that require state-selectivity to access specific energy windows in the local density of states will rely on precise amplitude calibration and waveform sampling in tunneling contact,^34^ which is part of ongoing work and beyond the scope of this publication.

## Conclusions and Outlook

In summary, we present a versatile THz pump–probe setup with continuous CEP control for lightwave-driven scanning probe microscopy at high repetition rates. Our system combines several key functionalities: (i) multi-MHz repetition rate laser pulses and a sensitive current preamplifier to detect light-driven tunneling currents, (ii) efficient single-cycle THz generation and divergence management, (iii) independent amplitude modulation, and (iv) continuous THz phase tuning in both the pump and probe arms. The latter is a key feature for precise access to the rich electronic structure of atomic quantum defects and other low-dimensional materials systems and paves the way for ultrafast scanning tunneling spectroscopy of complex materials.

Our system enables tip-induced voltages of 20 V at 1 MHz and 1 V at 41 MHz on a metallic substrate, consistent with far-field scaling and near-field amplitude calibration. These voltages estimated by PES inside the UHV chamber with a few-100 nm stand-off distance also apply in the tunneling regime of the STM.^35^ On the basis of frustrated total internal reflection in polymer prisms, we demonstrate continuous CEP tuning of THz pulses in the far and near field over a range of >0.7 π. Combined with a π phase flip provided by an additional mirror, we can access nearly the entire CEP space of 2π. Our method and modeling is applicable also to more advanced geometrical structures that control the phase of THz pulses via frustrated total internal reflection over larger intervals, e.g., a polymer rhombus with two internal reflections.

Continuous CEP tuning by frustrated total internal reflection is applicable to any THz-based far- and near-field technique requiring precise CEP control, including THz-STM,^1^ THz scanning tunneling spectroscopy,^32^ THz luminescence,^11^ THz action spectroscopy,^44^ THz scanning near-field optical microscopy (THz-SNOM),^45^ THz electro-optic modulation,^46^ and THz-based electron bunch compression.^47−49^ In particular, our method enables precise state-selective tunneling in THz-STM, providing a strong basis for the investigation of localized quantum states in low-dimensional materials systems.^50^

## Methods

### THz Pulse Generation

The THz generation and electro-optic detection of the transient waveform is fully scalable for a single-shot to 40 MHz repetition rate with an initial pulse energy of 50 to 0.5 μJ. Scanning the pulse delay in the infrared significantly increases the stability of our setup, achieving up to 2 ns relative delay between THz pulses. On the basis of the setup developed by Meyer et al.,^17^ we use two lenses and a transmission grating (Gitterwerk) to achieve efficient tilted-pulse-front phase matching at the LN crystal. To boost the THz conversion efficiency despite low pump pulse energies, we reduce the infrared spot size at the grating and at the LN crystal to about 500 and 300 μm, respectively. We use a 250 mm focal length for the first lens with an intermediate focus behind the grating and a 75 mm aspheric lens to image the grating to the LN crystal with 2:1 demagnification. This simple geometry of our THz generation reliably achieves THz conversion efficiencies up to 6 × 10^–4^ and intrinsically stabilizes minor pump beam pointing instabilities by design. Independent THz generation in two LN crystals has significant advantages as compared to sending both pump beams into a single LN crystal:^7^ (i) higher generation efficiency due to reduced thermal impact, (ii) independent CEP and amplitude control, and (iii) independent modulation of both THz arms at an intermediate focus. Electro-optic sampling with fundamental pulses enables scalable field detection at all repetition rates. Since only low THz frequencies couple efficiently to the near field, a deconvolution of the electro-optic signal as discussed by Sitnikov et al.^37^ showed no benefit to the presentation of the data.

### THz Power and Peak Field Estimate

We measure the THz power using a Gentec THz12D thermopile detector at the focus of a 100 mm PTFE lens. The specified power calibration uncertainty is <10%, as specified by the manufacturer. THz power and pulse energy stated in the manuscript account for 70% THz power transmissivity of the PTFE lens. Operating at high repetition rates with identical average power, we find that parasitic generation of continuous-wave thermal radiation and infrared scattering are negligible.

The THz peak field at the focus of electro-optic detection outside the STM is calibrated by calculating the inverse electro-optic effect. To do so, we divide the signal *S*(*t*) measured with the balanced detector by its full dynamic range Δ*S* and the electro-optic coefficient of our *d* = 1 mm-thick GaP crystal^51^*r*_eff_ = 0.97 pm/V. Accounting for a field reduction inside the GaP crystal^52^*G* ≈ 0.46, the THz far-field amplitude is approximately
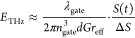
where λ_gate_ and *n*_gate_ are the wavelength and refractive index for the near-infrared gate pulse, respectively.

Due to different optical geometry and focusing conditions, the THz far-field amplitude inside the STM may slightly differ from this estimate. THz reflectivity and transmissivity of optical components suggest that approximately 30% of the THz peak field measured outside the STM reaches the tip–sample junction: two metallic mirrors with reflectivity *R* = 0.97 and three 2 mm c-cut sapphire windows with transmission *T* = 0.45. Due to additional clipping losses at the parabolic mirror inside the STM, we assume a total efficiency of 25%.

### Far- and Near-Field Sampling of the THz Waveform

We use a small fraction of the fundamental laser for electro-optic sampling of the THz far-field waveform in a 1 mm-thick GaP crystal outside the vacuum chamber. Sampling pulses are attenuated to 100 μW before the crystal, corresponding to a 100 pJ pulse energy at 1 MHz. The THz-induced polarization rotation in GaP is independent of sampling pulse energy and repetition rate and measured via ellipsometry using a quarter-wave plate, a 20° Wollaston prism, and an amplified balanced photodetector (Thorlabs PDB210A).

Approximately 200 mW of the fundamental laser is focused to a 1 mm-thick ß-BBO crystal (θ = 23.4°) and collinearly overlapped with the fundamental sampling beam using a harmonics combiner. At 1 MHz we obtain approximately 80 mW of SHG intensity corresponding to 40% conversion efficiency. To compensate a 1000 mm spherical mirror before the UHV chamber we prefocus the sampling pulse at a distance 1100 mm from this spherical mirror using a 500 mm lens. This allows compensating divergence and adapting the sampling pulse focal size on the STM tip using external optics. Inside the STM, we used a tungsten tip that was electrochemically etched from a 300 μm thick wire and indented into an Au crystal for tip forming purposes. We estimate the power of the sampling pulse at the STM tip–sample junction to 30% of the power measured outside the UHV chamber. For efficient PES at 1 MHz we use approximately 1–10 mW (1–10 nJ pulse energy) of SHG power at the STM tip. All near-field measurements are performed at liquid nitrogen temperature and <10^–10^ mbar UHV pressure.

### Analytical Calculations and Numerical Simulation

We calculate the reflected THz wave as an infinite sum of reflections between the prism and mirror surface on the basis of complex amplitude coefficients from the Fresnel equations. Losses in the dielectric are disregarded. Further, we treat the mirror as a perfect metal. Calculations are performed for a monochromatic wave with ν_0_ = 0.8 THz, which is the central frequency of the THz pulse in our experiments. Instead of a CEP value, we show the relative shift of a single field maximum of the reflected wave as a function of mirror–prism distance.

Finite difference time domain simulations are performed with commercial Lumerical (Ansys) software. Input Gaussian pulses have a central frequency of 0.8 THz and a spectral width of 0.5 THz. The dielectric prism is considered nondispersive with a fixed refractive index of 1.43. The movable mirror is regarded as a perfect metal. The simulation is performed in two-dimension (2D) and perfectly matched layers truncate the physical domain. Mesh cells are reduced to a 2 μm size to achieve convergence of the simulation.

## Supporting Information Available

Details on THz generation and amplitude control; additional information on prism performance and carrier-envelope phase fitting; power and distance dependence of photoemission sampling; THz amplitude calibration in the tunneling contact with Au(111); and THz beam divergence management (PDF)

## References

[ref1] Cocker T. L.; Peller D.; Yu P.; Repp J.; Huber R. Tracking the Ultrafast Motion of a Single Molecule by Femtosecond Orbital Imaging. Nature 2016, 539 (7628), 263–267. 10.1038/nature19816. 27830788 PMC5597038

[ref2] Ludwig M.; Aguirregabiria G.; Ritzkowsky F.; Rybka T.; Marinica D. C.; Aizpurua J.; Borisov A. G.; Leitenstorfer A.; Brida D. Sub-Femtosecond Electron Transport in a Nanoscale Gap. Nat. Phys. 2020, 16 (3), 341–345. 10.1038/s41567-019-0745-8.

[ref3] Arashida Y.; Mogi H.; Ishikawa M.; Igarashi I.; Hatanaka A.; Umeda N.; Peng J.; Yoshida S.; Takeuchi O.; Shigekawa H. Subcycle Mid-Infrared Electric-Field-Driven Scanning Tunneling Microscopy with a Time Resolution Higher Than 30 Fs. ACS Photonics 2022, 9, 3156–3164. 10.1021/acsphotonics.2c00995.

[ref4] Luo Y.; Martin-Jimenez A.; Neubrech F.; Liu N.; Garg M. Synthesis and Direct Sampling of Single-Cycle Light Transients by Electron Tunneling in a Nanodevice. ACS Photonics 2023, 10 (8), 2866–2873. 10.1021/acsphotonics.3c00584.

[ref5] Cocker T. L.; Jelic V.; Gupta M.; Molesky S. J.; Burgess J. A. J.; Reyes G. D. L.; Titova L. V.; Tsui Y. Y.; Freeman M. R.; Hegmann F. A. An Ultrafast Terahertz Scanning Tunnelling Microscope. Nat. Photonics 2013, 7 (8), 620–625. 10.1038/nphoton.2013.151.

[ref6] Yoshida S.; Hirori H.; Tachizaki T.; Yoshioka K.; Arashida Y.; Wang Z.-H.; Sanari Y.; Takeuchi O.; Kanemitsu Y.; Shigekawa H. Subcycle Transient Scanning Tunneling Spectroscopy with Visualization of Enhanced Terahertz Near Field. ACS Photonics 2019, 6 (6), 1356–1364. 10.1021/acsphotonics.9b00266.

[ref7] Abdo M.; Sheng S.; Rolf-Pissarczyk S.; Arnhold L.; Burgess J. A. J.; Isobe M.; Malavolti L.; Loth S. Variable Repetition Rate THz Source for Ultrafast Scanning Tunneling Microscopy. ACS Photonics 2021, 8 (3), 702–708. 10.1021/acsphotonics.0c01652. 33763504 PMC7976605

[ref8] Yoshida S.; Arashida Y.; Hirori H.; Tachizaki T.; Taninaka A.; Ueno H.; Takeuchi O.; Shigekawa H. Terahertz Scanning Tunneling Microscopy for Visualizing Ultrafast Electron Motion in Nanoscale Potential Variations. ACS Photonics 2021, 8 (1), 315–323. 10.1021/acsphotonics.0c01572.

[ref9] Liu S.; Hammud A.; Hamada I.; Wolf M.; Müller M.; Kumagai T. Nanoscale Coherent Phonon Spectroscopy. Sci. Adv. 2022, 8 (42), eabq5682 10.1126/sciadv.abq5682. 36269832 PMC9586471

[ref10] Plankl M.; Faria Junior P. E.; Mooshammer F.; Siday T.; Zizlsperger M.; Sandner F.; Schiegl F.; Maier S.; Huber M. A.; Gmitra M.; Fabian J.; Boland J. L.; Cocker T. L.; Huber R. Subcycle Contact-Free Nanoscopy of Ultrafast Interlayer Transport in Atomically Thin Heterostructures. Nat. Photonics 2021, 15 (8), 594–600. 10.1038/s41566-021-00813-y.

[ref11] Kimura K.; Morinaga Y.; Imada H.; Katayama I.; Asakawa K.; Yoshioka K.; Kim Y.; Takeda J. Terahertz-Field-Driven Scanning Tunneling Luminescence Spectroscopy. ACS Photonics 2021, 8, 982–987. 10.1021/acsphotonics.0c01755.

[ref12] Lloyd-Hughes J.; Oppeneer P. M.; dos Santos T. P.; Schleife A.; Meng S.; Sentef M. A.; Ruggenthaler M.; Rubio A.; Radu I.; Murnane M.; Shi X.; Kapteyn H.; Stadtmüller B.; Dani K. M.; da Jornada F. H.; Prinz E.; Aeschlimann M.; Milot R. L.; Burdanova M.; Boland J.; Cocker T.; Hegmann F. The 2021 Ultrafast Spectroscopic Probes of Condensed Matter Roadmap. J. Phys.: Condens. Matter 2021, 33 (35), 353001 10.1088/1361-648X/abfe21. 33951618

[ref13] Wang L.; Xia Y.; Ho W. Atomic-Scale Quantum Sensing Based on the Ultrafast Coherence of an H2Molecule in an STM Cavity. Science 2022, 376 (6591), 401–405. 10.1126/science.abn9220. 35446636

[ref14] Fülöp J. A.; Pálfalvi L.; Almási G.; Hebling J. Design of High-Energy Terahertz Sources Based on Optical Rectification. Opt. Express 2010, 18 (12), 12311–12327. 10.1364/OE.18.012311. 20588357

[ref15] Tsarev M. V.; Ehberger D.; Baum P. High-Average-Power, Intense THz Pulses from a LiNbO3 Slab with Silicon Output Coupler. Appl. Phys. B 2016, 122 (2), 30 10.1007/s00340-015-6315-6.

[ref16] Fülöp J. A.; Tzortzakis S.; Kampfrath T. Laser-Driven Strong-Field Terahertz Sources. Adv. Opt. Mater. 2020, 8 (3), 1900681 10.1002/adom.201900681.

[ref17] Meyer F.; Vogel T.; Ahmed S.; Saraceno C. J. Single-Cycle, MHz Repetition Rate THz Source with 66 mW of Average Power. Opt. Lett. 2020, 45 (9), 2494–2497. 10.1364/OL.386305. 32356799

[ref18] Kuttruff J.; Tsarev M. V.; Baum P. Jitter-Free Terahertz Pulses from LiNbO _3_. Opt. Lett. 2021, 46 (12), 2944–2947. 10.1364/OL.430507. 34129580

[ref19] Guiramand L.; Nkeck J. E.; Ropagnol X.; Ozaki T.; Blanchard F. Near-Optimal Intense and Powerful Terahertz Source by Optical Rectification in Lithium Niobate Crystal. Photon. Res. 2022, 10 (2), 340–346. 10.1364/PRJ.428418.

[ref20] Kroh T.; Rohwer T.; Zhang D.; Demirbas U.; Cankaya H.; Hemmer M.; Hua Y.; Zapata L. E.; Pergament M.; Kärtner F. X.; Matlis N. H. Parameter Sensitivities in Tilted-Pulse-Front Based Terahertz Setups and Their Implications for High-Energy Terahertz Source Design and Optimization. Opt. Express 2022, 30 (14), 24186–24206. 10.1364/OE.457773. 36236979

[ref21] Ammerman S. E.; Jelic V.; Wei Y.; Breslin V. N.; Hassan M.; Everett N.; Lee S.; Sun Q.; Pignedoli C. A.; Ruffieux P.; Fasel R.; Cocker T. L. Lightwave-Driven Scanning Tunnelling Spectroscopy of Atomically Precise Graphene Nanoribbons. Nat. Commun. 2021, 12 (1), 6794 10.1038/s41467-021-26656-3. 34815398 PMC8611099

[ref22] Gingras L.; Cui W.; Schiff-Kearn A. W.; Ménard J.-M.; Cooke D. G. Active Phase Control of Terahertz Pulses Using a Dynamic Waveguide. Opt. Express 2018, 26 (11), 13876–13882. 10.1364/OE.26.013876. 29877433

[ref23] Herter A.; Shams-Ansari A.; Settembrini F. F.; Warner H. K.; Faist J.; Lončar M.; Benea-Chelmus I.-C. Terahertz Waveform Synthesis in Integrated Thin-Film Lithium Niobate Platform. Nat. Commun. 2023, 14 (1), 11 10.1038/s41467-022-35517-6. 36599838 PMC9812977

[ref24] Kawada Y.; Yasuda T.; Takahashi H. Carrier Envelope Phase Shifter for Broadband Terahertz Pulses. Opt. Lett. 2016, 41 (5), 986–989. 10.1364/OL.41.000986. 26974097

[ref25] Yoshioka K.; Katayama I.; Arashida Y.; Ban A.; Kawada Y.; Konishi K.; Takahashi H.; Takeda J. Tailoring Single-Cycle Near Field in a Tunnel Junction with Carrier-Envelope Phase-Controlled Terahertz Electric Fields. Nano Lett. 2018, 18 (8), 5198–5204. 10.1021/acs.nanolett.8b02161. 30028952

[ref26] Ruffin A. B.; Rudd J. V.; Whitaker J. F.; Feng S.; Winful H. G. Direct Observation of the Gouy Phase Shift with Single-Cycle Terahertz Pulses. Phys. Rev. Lett. 1999, 83 (17), 3410–3413. 10.1103/PhysRevLett.83.3410.

[ref27] Feng S.; Winful H. G. Physical Origin of the Gouy Phase Shift. Opt. Lett. 2001, 26 (8), 485–487. 10.1364/OL.26.000485. 18040360

[ref28] Lindner F.; Paulus G. G.; Walther H.; Baltuška A.; Goulielmakis E.; Lezius M.; Krausz F. Gouy Phase Shift for Few-Cycle Laser Pulses. Phys. Rev. Lett. 2004, 92 (11), 113001 10.1103/PhysRevLett.92.113001. 15089129

[ref29] Yoshioka K.; Katayama I.; Minami Y.; Kitajima M.; Yoshida S.; Shigekawa H.; Takeda J. Real-Space Coherent Manipulation of Electrons in a Single Tunnel Junction by Single-Cycle Terahertz Electric Fields. Nat. Photonics 2016, 10 (12), 762–765. 10.1038/nphoton.2016.205.

[ref30] Li T.; Quan B.; Fang G.; Wang T. Flexible THz Carrier-Envelope Phase Shifter Based on Metamaterials. Adv. Opt. Mater. 2022, 10, 2200541 10.1002/adom.202200541.

[ref31] Korytin A. I.; Lavrent’ev S. A.; Mishakin S. V. New Method of Control over the Phase of an Ultrashort Electromagnetic Pulse under Frustrated Total Internal Reflection Conditions. JETP Lett. 2007, 86 (7), 451–453. 10.1134/S0021364007190046.

[ref32] Ammerman S. E.; Wei Y.; Everett N.; Jelic V.; Cocker T. L. Algorithm for Subcycle Terahertz Scanning Tunneling Spectroscopy. Phys. Rev. B 2022, 105 (11), 115427 10.1103/PhysRevB.105.115427.

[ref33] Katayama I.; Kimura K.; Imada H.; Kim Y.; Takeda J. Investigation of Ultrafast Excited-State Dynamics at the Nanoscale with Terahertz Field-Induced Electron Tunneling and Photon Emission. J. Appl. Phys. 2023, 133 (11), 110903 10.1063/5.0144218.

[ref34] Peller D.; Roelcke C.; Kastner L. Z.; Buchner T.; Neef A.; Hayes J.; Bonafé F.; Sidler D.; Ruggenthaler M.; Rubio A.; Huber R.; Repp J. Quantitative Sampling of Atomic-Scale Electromagnetic Waveforms. Nat. Photonics 2021, 15 (2), 143–147. 10.1038/s41566-020-00720-8.

[ref35] Müller M.; Martín Sabanés N.; Kampfrath T.; Wolf M. Phase-Resolved Detection of Ultrabroadband THz Pulses inside a Scanning Tunneling Microscope Junction. ACS Photonics 2020, 7 (8), 2046–2055. 10.1021/acsphotonics.0c00386. 32851116 PMC7441495

[ref36] Wulf F.; Hoffmann M.; Saraceno C. J. Analysis of THz Generation Using the Tilted-Pulse-Front Geometry in the Limit of Small Pulse Energies and Beam Sizes. Opt. Express 2021, 29 (12), 18889–18904. 10.1364/OE.426228. 34154135

[ref37] Sitnikov D. S.; Romashevskiy S. A.; Ovchinnikov A. V.; Chefonov O. V.; Savel’ev A. B.; Agranat M. B. Estimation of THz Field Strength by an Electro-Optic Sampling Technique Using Arbitrary Long Gating Pulses. Laser Phys. Lett. 2019, 16 (11), 115302 10.1088/1612-202X/ab4d56.

[ref38] Doppagne B.; Chong M. C.; Bulou H.; Boeglin A.; Scheurer F.; Schull G. Electrofluorochromism at the Single-Molecule Level. Science 2018, 361 (6399), 251–255. 10.1126/science.aat1603. 30026221

[ref39] Bichon J.; Pillet A.; Sklia A.; Petitprez D.; Peretti R.; Eliet S. In Complex Refractive Index Determination of PTFE, TPX and Polypropylene Windows for TeraHertz Broadband Spectroscopy, 022 47th International Conference on Infrared, Millimeter and Terahertz Waves (IRMMW-THz); IEEE: Delft, Netherlands, 2022.

[ref40] Islam M. S.; Cordeiro C. M. B.; Nine M. J.; Sultana J.; Cruz A. L. S.; Dinovitser A.; Ng B. W.-H.; Ebendorff-Heidepriem H.; Losic D.; Abbott D. Experimental Study on Glass and Polymers: Determining the Optimal Material for Potential Use in Terahertz Technology. IEEE Access 2020, 8, 97204–97214. 10.1109/ACCESS.2020.2996278.

[ref41] Sultanova N.; Kasarova S.; Nikolov I. Dispersion Properties of Optical Polymers. Acta Phys. Polym., A 2009, 116 (4), 585–587. 10.12693/APhysPolA.116.585.

[ref42] Wimmer L.; Herink G.; Solli D. R.; Yalunin S. V.; Echternkamp K. E.; Ropers C. Terahertz Control of Nanotip Photoemission. Nat. Phys. 2014, 10 (6), 432–436. 10.1038/nphys2974.

[ref43] Jelic V.; Iwaszczuk K.; Nguyen P. H.; Rathje C.; Hornig G. J.; Sharum H. M.; Hoffman J. R.; Freeman M. R.; Hegmann F. A. Ultrafast Terahertz Control of Extreme Tunnel Currents through Single Atoms on a Silicon Surface. Nat. Phys. 2017, 13 (6), 591–598. 10.1038/nphys4047.

[ref44] Peller D.; Kastner L. Z.; Buchner T.; Roelcke C.; Albrecht F.; Moll N.; Huber R.; Repp J. Sub-Cycle Atomic-Scale Forces Coherently Control a Single-Molecule Switch. Nature 2020, 585 (7823), 58–62. 10.1038/s41586-020-2620-2. 32879499

[ref45] Cocker T. L.; Jelic V.; Hillenbrand R.; Hegmann F. A. Nanoscale Terahertz Scanning Probe Microscopy. Nat. Photonics 2021, 15 (8), 558–569. 10.1038/s41566-021-00835-6.

[ref46] Ren Z.; Xu J.; Liu J.; Li B.; Zhou C.; Sheng Z. Active and Smart Terahertz Electro-Optic Modulator Based on VO _2_ Structure. ACS Appl. Mater. Interfaces 2022, 14 (23), 26923–26930. 10.1021/acsami.2c04736. 35652202

[ref47] Kealhofer C.; Schneider W.; Ehberger D.; Ryabov A.; Krausz F.; Baum P. All-Optical Control and Metrology of Electron Pulses. Science 2016, 352 (6284), 429–433. 10.1126/science.aae0003. 27102476

[ref48] Ehberger D.; Mohler K. J.; Vasileiadis T.; Ernstorfer R.; Waldecker L.; Baum P. Terahertz Compression of Electron Pulses at a Planar Mirror Membrane. Phys. Rev. Appl. 2019, 11 (2), 024034 10.1103/PhysRevApplied.11.024034.

[ref49] Snively E. C.; Othman M. A. K.; Kozina M.; Ofori-Okai B. K.; Weathersby S. P.; Park S.; Shen X.; Wang X. J.; Hoffmann M. C.; Li R. K.; Nanni E. A. Femtosecond Compression Dynamics and Timing Jitter Suppression in a THz-Driven Electron Bunch Compressor. Phys. Rev. Lett. 2020, 124 (5), 054801 10.1103/PhysRevLett.124.054801. 32083908

[ref50] Robinson J. A.; Schuler B. Engineering and Probing Atomic Quantum Defects in 2D Semiconductors: A Perspective. Appl. Phys. Lett. 2021, 119 (14), 140501 10.1063/5.0065185.

[ref51] Nelson D. F.; Turner E. H. Electro-Optic and Piezoelectric Coefficients and Refractive Index of Gallium Phosphide. J. Appl. Phys. 1968, 39 (7), 3337–3343. 10.1063/1.1656779.

[ref52] Wu Q.; Zhang X.-C. 7 Terahertz Broadband GaP Electro-Optic Sensor. Appl. Phys. Lett. 1997, 70 (14), 1784–1786. 10.1063/1.118691.

