# Supplementary material for: Efficient and Continuous Carrier-Envelope Phase Control for Terahertz Lightwave-Driven Scanning Probe Microscopy
Source: ACS Photonics. 2023 Oct 11;10(11):3888–95. doi: 10.1021/acsphotonics.3c00555 (PMC10655500; doi:10.1021/acsphotonics.3c00555)
Supplement: Supplementary file 1 — ph3c00555_si_001.pdf [file ph3c00555_si_001.pdf]

Supporting Information

Efficient and Continuous Carrier-Envelope Phase Control for Terahertz Lightwave-Driven Scanning Probe Microscopy

Jonas Allerbeck[1*], Joel Kuttruff[2], Laric Bobzien[1], Lysander Huberich[1], Maxim Tsarev[2], Bruno Schuler[1+]

1. nanotech@surfaces Laboratory, Empa, Swiss Federal Laboratories for Materials Science and Technology, Überlandstrasse 129, 8600 Dübendorf, Switzerland

2. Department of Physics, University of Konstanz, Universitätsstrasse 10, 78464 Konstanz, Germany

Corresponding authors: *jonas.allerbeck@empa.ch, +bruno.schuler@empa.ch

9 pages, 7 figures

1. **Details on the THz Generation and Amplitude Control**
2. **Prism Performance and Carrier-Envelope Phase Fitting**
3. **Power and Tip–Sample Distance Scaling of Photoemission Sampling**
4. **THz Amplitude Calibration in Tunneling Feedback on Au(111)**
5. **THz Beam Divergence Management**

1. Details on the THz Generation and Amplitude Control

The geometric configuration and optical components of both THz branches in our setup are identical apart from minor alignment differences. Figure S1 compares the generation efficiency of the THz2 line in analogy to Figure 2 of the manuscript. In the current configuration, the THz1 branch reaches slightly higher conversion efficiency at higher repetition rates in comparison to THz2. For both branches, the THz field amplitude scales linear with pump pulse energy up to 10 μJ.

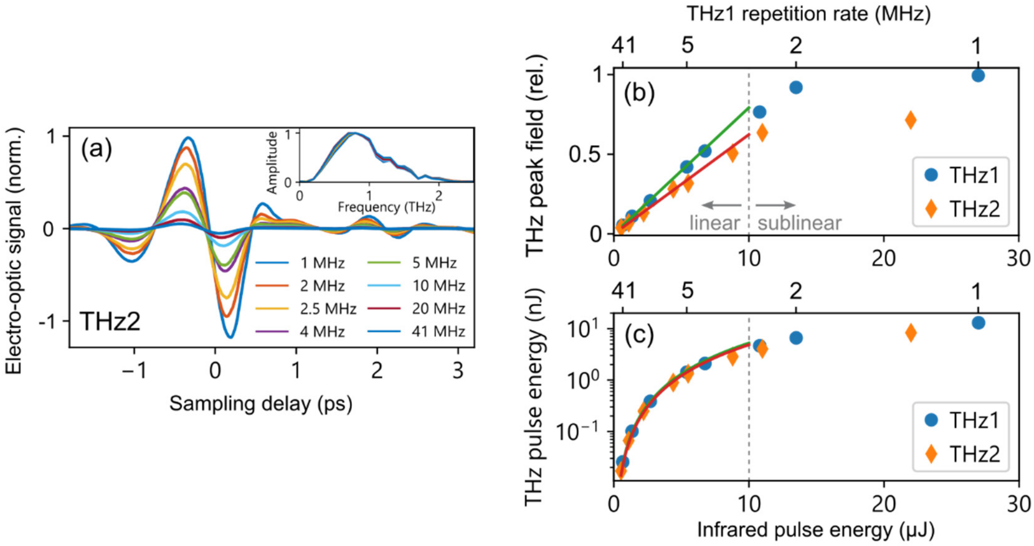

Figure S1. Performance of THz generation. (a) Field transients of THz branch 2 (THz2) at various repetition rates. (b) THz peak field measured in EOS as a function of repetition rate and pump pulse energy. (c) Corresponding THz pulse energy as measured with a thermal detector (Gentec THz12D) directly after generation and collimation.

Figure S2a shows the noncollinear THz beam recombination and the overlap with sampling pulses or visible alignment lasers using an indium tin oxide (ITO) coated THz beam combiner. The THz waveform as generated by the lithium niobate (LN) crystal have almost identical shape in both arms. Panel (c) shows the electro-optic signal as a function of sampling and relative THz delay, showing linear interference of both pulses. A long range scan in panel (d) at 6 ps THz-THz delay shows no reflexes or trailing pulses other than those observed through multiple reflections in the 1 mm-thick GaP crystal used for electro-optic detection. We purge our THz setup with dry air to reduce water vapor absorption. Residual tailing field oscillations seen in panel (d) are sufficiently weak and do not limit the experiment.

We use two different methods to control the amplitude of THz pulses illustrated in Figure S3a. In branch THz1, a pair of wiregrid polarizers enables precise control of the field amplitude. Panel (b) shows the THz waveform as a function of rotation angle θ of the first polarizer. At relative field amplitude >10 %, the amplitude scaling is perfectly linear with relative field amplitude as

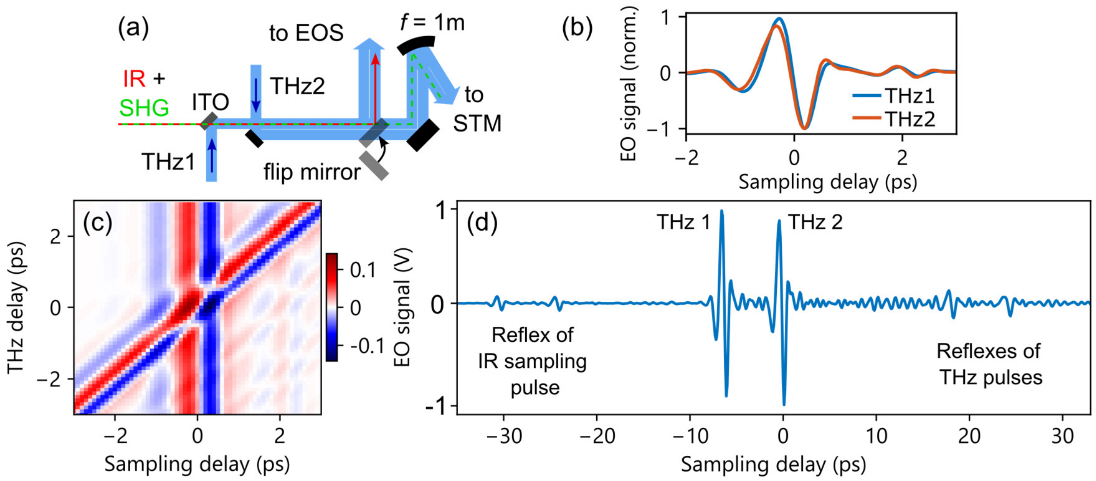

Figure S2. THz pump-probe characterized via electro-optic sampling. (a) Noncollinear overlap of independent THz beams. (b) THz waveform obtained from individual generation arms. (c) Combined electro-optic (EO) signal of both THz pulses as a function of the relative delay of the THz1 pulse. (e) EO signal of both THz pulses with 6 ps delay showing reflexes in the 1 mm-thick GaP detection crystal.

calculated by Lambert-Beer's law $E = E_0\cos^2(\theta)$. For the THz2 branch we use a half-wave plate to rotate the polarization of infrared pump pulses before THz generation, which is a much simpler and cost-efficient solution. Since only the projected pump pulse polarization parallel to the nonlinear axis of the lithium niobate (LN) crystal contributes to THz generation, this method does not require splitting of the polarization components. For pump pulse energies <10 µJ, hence at repetition rates >3 MHz, the peak field amplitude of generated THz pulses scales linearly with IR pump power. In this regime the field amplitude is given by $E = E_0\cos^2(2\alpha)$, where α is the waveplate rotation angle.

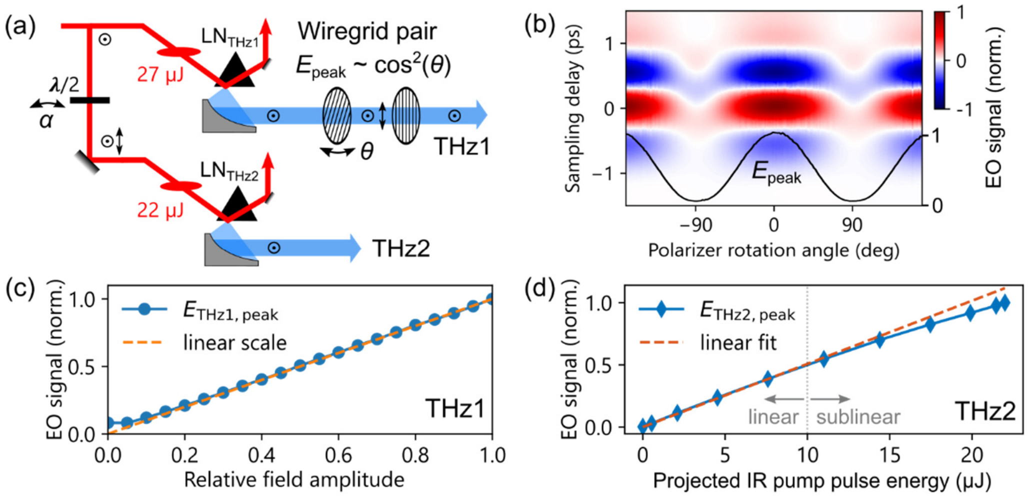

Figure S3. THz amplitude control. (a) Optical setup. THz1 uses a pair of wiregrid polarizes to attenuate precisely the THz pulses after optical rectification, while in THz2 we rotate the infrared (IR) pump pulse polarization using a half-wave plate. (b) THz1 field profile as a function of the wiregrid polarizer rotation. Below 10% field amplitude attenuation is imperfect and cannot be used in experiments. (c,d) Scaling of the field amplitude (EO signal) of THz1 and THz2 as a function of relative field amplitude or projected IR pump pulse energy (polarization parallel to the LN optical axis) respectively.

2. Prism Performance and Carrier-Envelope Phase Fitting

Figure S4 compares the performance of PTFE and Zeonex prisms with respect to a plain Ag mirror instead of the prism. Panels (a) and (b) show the amplitude transmission in time and frequency domain, which is slightly redshifted for the PTFE prism owing to stronger absorption of high frequency components[1]**.** The dashed lines in panel (b) indicate the spectral amplitude transmission as a guide to the eye. The relative phase shift induced by the PTFE prism can be characterized by two methods: (i) tracking the absolute phase delay τ, and (ii) fitting an analytic waveform to the measured THz transient. To do this we use the python *scipy.curvefit* least-squares algorithm to fit the field profile of experimental and numeric data in a 2 ps interval around the center of the pulse envelope. All fit parameter are open within reasonable limits. The fit function is

$$E(t) = E_0 \cos(2\pi(t - t_0)\nu + \phi_0) \exp\left(-\left(\frac{t-t_0}{\tau_{\mathrm{fwhm}}/(2\sqrt{ln2})}\right)^2\right).$$

E_0: field amplitude, t: sampling time and offset t_0, ν: central THz frequency, ϕ_0 carrier-envelope phase offset, τ_{fwhm}: pulse duration.

The maximum phase shift achieved with the PTFE prism and measured with this method is $\Delta\phi_{\max} \approx 0.76\,\pi$, corresponding to the phase difference of 0 mm (yellow curve) and 2 mm mirror-prism distance (blue curve) in between which the peak field maximum shifts by $\tau \approx 0.33$ ps.

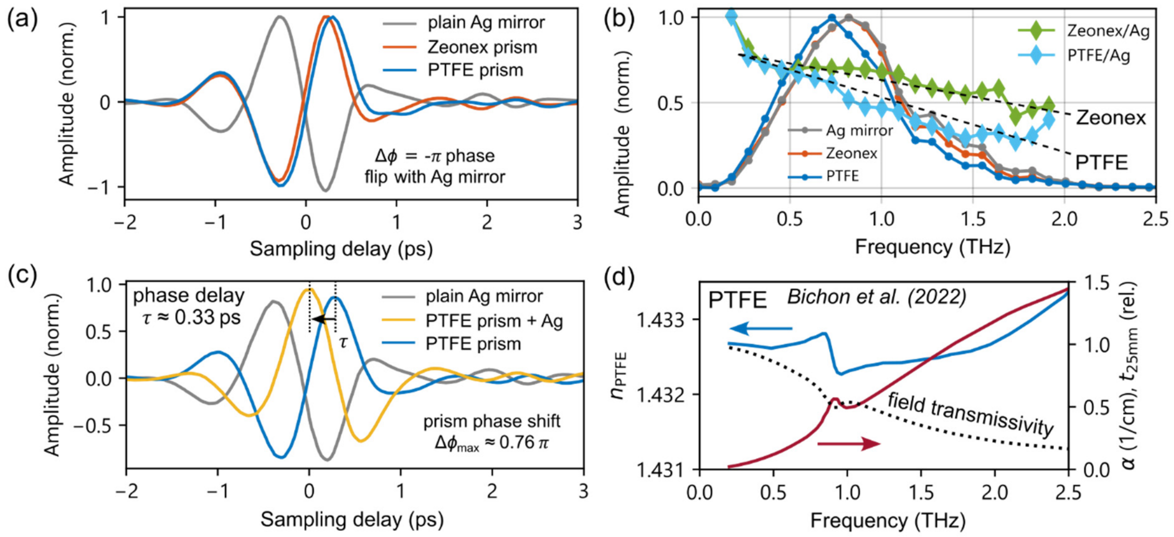

Figure S4: Prism transmissivity. (a) THz waveform measured with a plain Ag mirror and PTFE or Zeonex prism (mirror retracted). (b) Normalized amplitude spectrum of the measurements in (a). Relative amplitude with respect to the plain mirror reflects the spectral amplitude transmissivity of the material. Black dashed lines guide the eye. (c) THz waveform transmitted by the PTFE prism with and without an Ag mirror touching the prism surface (0 mm distance). The THz CEP shift induced by the PTFE prism is estimated by fitting the transient waveform. (d) Spectral refractive index and absorption coefficient of PTFE as published by *Bichon et al.*[1] The dotted line indicates relative field transmissivity for 25 mm material passage.

3. Power and Tip–Sample Distance Scaling of Photoemission Sampling

At high THz field strength, for different alignment conditions, i.e. tip apex or tip shaft, or different tip-sample distances, waveform distortions must be considered. To ensure the validity of our experiments presented throughout the manuscript we performed power dependent and tip–sample distance dependent measurements of the near-field waveform[2]. For reasonable experimental configurations, Figure S5 indeed shows variations of the THz amplitude as calibrated by photoemission sampling, however changes of the normalized waveform remain negligible. In particular at high pulse energies of the photoemission sampling pulse, the THz amplitude reduces significantly due to space charging effects that manifest in a saturation of the power scaling of the DC photoemission current $I_{10\mathrm{V}}^{\mathrm{DC}}$ in Fig S5b. The reduction of PES calibrated voltage at lower pulse energy might be a saturation of the THz modulated current related to the short duty cycle of THz pulses. In turn, this indicates that the THz amplitude is underestimated for measurements with 4 nJ sampling pulse energy. Previous studies found that the THz voltage remains independent for small tip-sample distances[2–4]; however, as shown in Figure S5c-d, the PES signal varies on a few-100 nm scale due to interference of the gate pulse within the tip–sample junction

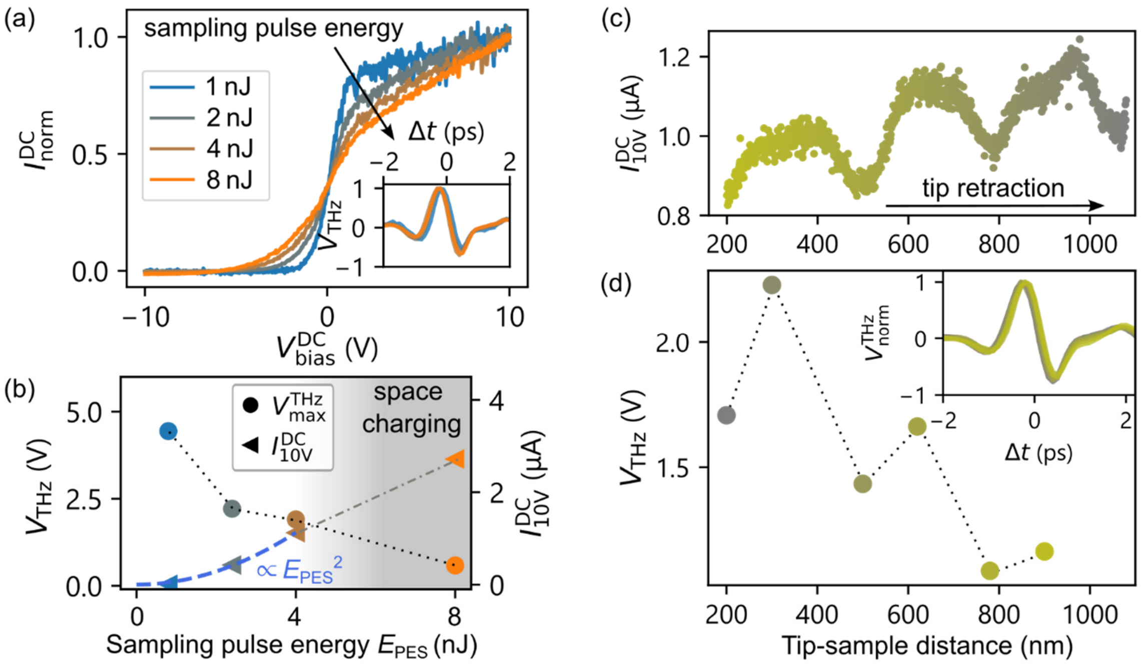

Figure S5. PES power and distance dependence. (a) I-V curves measured at different sampling pulse energies at 1 MHz. All curves are normalized at V_{bias} = 10 V. The inset shows the normalized near-field waveform for different sampling pulse energies, indicating negligible phase changes. (b) Peak THz field and DC PES current at 10 V measured as a function of sampling pulse energy. At high sampling power space charging effects lead to a reduced power scaling indicated by the dash-dotted line. (c) DC PES current as a function of tip-sample distance. PES current oscillations with a 300 nm modulation period may result from an interference modulation of sampling pulse reflexes. (d) Peak THz voltage measured at different tip-sample distances. A dotted line in panels (b) and (d) serves a guide to the eye. We note that THz voltages in panels (b) and (d) correspond to attenuated THz pulses.

(tungsten tip and Au(111) sample). In the scope of this manuscript, PES provides a qualitative measurement of the near-field waveform that consistently reproduces relative phase changes within each experimental configuration.

4. THz Amplitude Calibration in Tunneling Feedback on Au(111)

We benchmark the amplitude calibration performed via PES with an independent method (similar to reference[5], Figure S3) in tunneling contact with an Au(111) crystal. Figure S6a shows the rectified charge per THz pulse Q_{THz} as a function of relative THz field amplitude for different CEP modulated waveforms. As indicated by the inset, this curve is symmetric in case of a bipolar waveform (red) and asymmetric for a unipolar waveform (purple). The CEP shifter allows to continuously sweep between these configurations. The asymmetry of charge rectification measured with a unipolar waveform stems from an asymmetry of the density of states. To calibrate

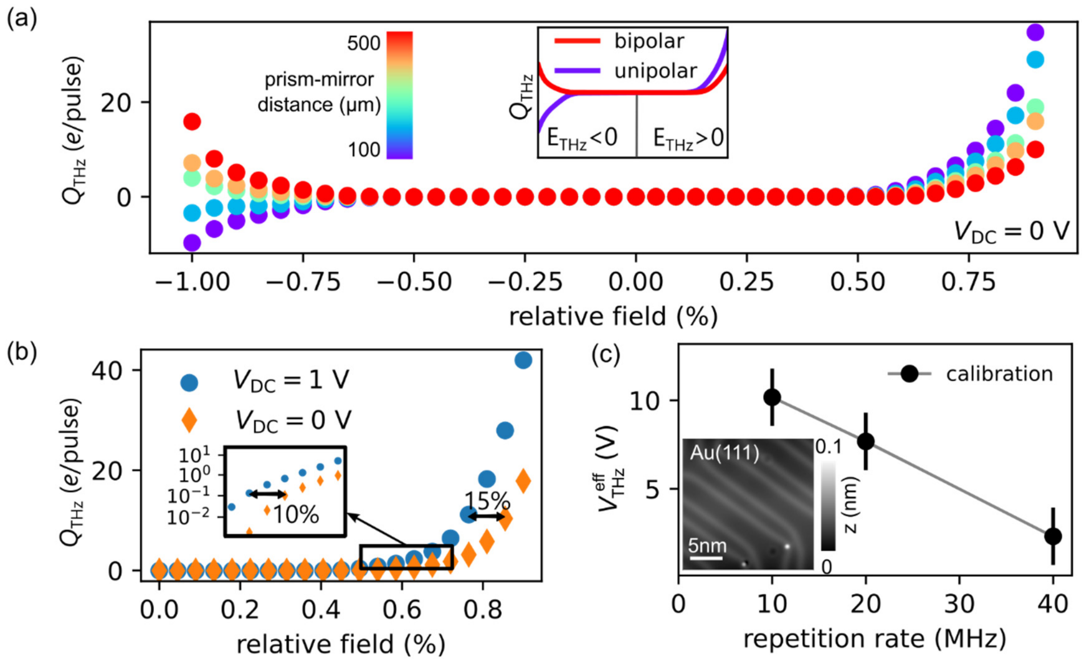

Figure S6. THz amplitude calibration in tunneling feedback on Au(111). (a) Rectified charge Q_{THz} measured as a function of relative THz field for both pulse polarities and different CEP modulated waveform types. The inset emphasizes the expected qualitative scaling of the curves of for bipolar and unipolar waveforms (in color). (b) Rectified charge for a unipolar waveform measured at 0 V and 1 V dc bias using 10 MHz repetition rate. Arrows indicate the horizontal shift between both curves used for the relative field-to-voltage calibration. The inset emphasizes the shift at lower rectification levels. (c) THz effective voltage calibration at multi-MHz repetition rates in tunneling contact. Vertical lines indicate the error bar. The inset shows STM topography of the Au(111) surface with herringbone reconstruction and an Au adatom. The DC tunneling setpoint for all measurements is V_{DC} = 50 mV, I_{DC} = 200 pA.

relative field to effective THz voltage, we perform the same measurement for different DC bias voltage as shown in panel (b). The horizontal shift of the curves calibrates relative field to the dc offset. The inset highlights the shift at rectification levels below one electron/pulse. In this example, the THz peak field calibration 1 V/0.1 = 10 V for low rectification levels (<1 electron/pulse) and slightly weaker at high currents. Figure S6c shows the calibration performed at different repetition rates with approximately linear scaling. The calibration was not performed at repetition rates < 10 MHz because large THz amplitudes beyond 10 V induced instabilities and tip changes when the tip is in tunneling contact with the sample. The values estimated here even exceed the estimate based on PES shown in Figure 2c of the manuscript. Deviations are related to a different mesoscopic shape of the STM tip and optics alignment. The inset in panel (c) shows the STM topography of the herringbone reconstruction of the Au(111) surface.

5. THz Beam Divergence Management

Figure S7a shows the complete 2.8 m-long THz beam path with all optical components from the lithium niobate (LN) crystal to the STM tip. For readability, focusing reflective components are depicted as lenses. Black bars mark the clear aperture of optical components at various positions in the setup. Panel (b) shows the measured 90 % beam diameter (circles) at different positions of the setup. Blue lines show a fit to the data points estimating the typical intrinsic divergence given by the THz Rayleigh length. Yellow lines are calculations for different focusing optics to estimate the beam diameter at positions where it cannot be measured. Shaded regions of fits and calculations take into account the spectral bandwidth of THz pulses.

A *f* = 1000 mm spherical mirror reduces the THz beam diameter towards the vacuum chamber to match the aperture of 25 mm optics inside the STM without an intermediate focus. Since the focus of the THz at the tip-sample junction is on the order of 1 mm, this has negligible impact on the near-field coupling but instead boosts the transmitted power.

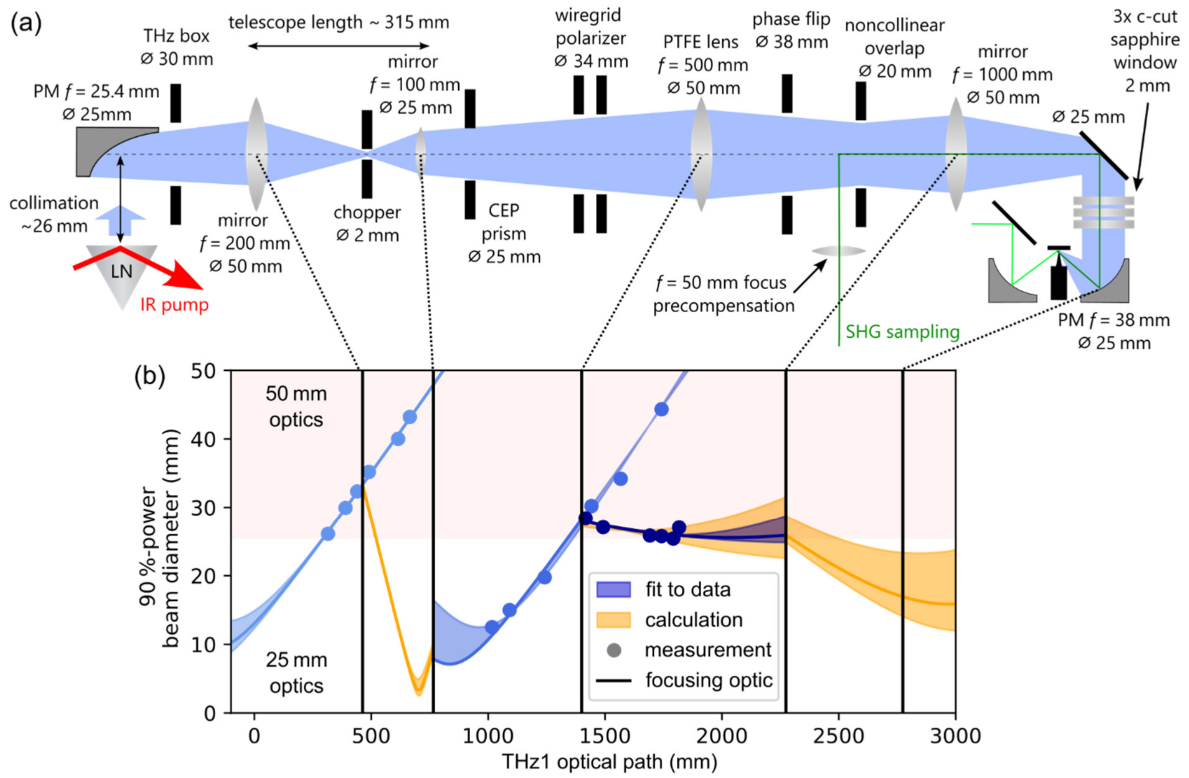

Figure S7. THz beam divergence control. (a) Conceptual illustration of the complete THz1 path from generation (LN at 0 mm) to the STM (at 2800 mm). Black bars indicate the clear aperture of various optical components. For simplicity, spherical mirrors are depicted as lenses. (b) Measurement (blue) and calculation of THz divergence on the basis of Gaussian optics. The shaded areas indicate broadband frequency of the single-cycle pulses in the calculation.

References

(1) Bichon, J.; Pillet, A.; Sklia, A.; Petitprez, D.; Peretti, R.; Eliet, S. Complex Refractive Index Determination of PTFE, TPX and Polypropylene Windows for TeraHertz Broadband Spectroscopy. In *2022 47th International Conference on Infrared, Millimeter and Terahertz Waves (IRMMW-THz)*; IEEE: Delft, Netherlands, 2022; pp 1–2. https://doi.org/10.1109/IRMMW-THz50927.2022.9895520.

(2) Müller, M.; Martín Sabanés, N.; Kampfrath, T.; Wolf, M. Phase-Resolved Detection of Ultrabroadband THz Pulses inside a Scanning Tunneling Microscope Junction. *ACS Photonics* **2020**, *7* (8), 2046–2055. https://doi.org/10.1021/acsphotonics.0c00386.

(3) Jelic, V.; Iwaszczuk, K.; Nguyen, P. H.; Rathje, C.; Hornig, G. J.; Sharum, H. M.; Hoffman, J. R.; Freeman, M. R.; Hegmann, F. A. Ultrafast Terahertz Control of Extreme Tunnel Currents through Single Atoms on a Silicon Surface. *Nature Phys* **2017**, *13* (6), 591–598. https://doi.org/10.1038/nphys4047.

(4) Chen, S.; Shi, W.; Ho, W. Single-Molecule Continuous-Wave Terahertz Rectification Spectroscopy and Microscopy. *Nano Lett.* **2023**, *23* (7), 2915–2920. https://doi.org/10.1021/acs.nanolett.3c00271.

(5) Yoshida, S.; Arashida, Y.; Hirori, H.; Tachizaki, T.; Taninaka, A.; Ueno, H.; Takeuchi, O.; Shigekawa, H. Terahertz Scanning Tunneling Microscopy for Visualizing Ultrafast Electron Motion in Nanoscale Potential Variations. *ACS Photonics* **2021**, *8* (1), 315–323. https://doi.org/10.1021/acsphotonics.0c01572.
